# Does a Compatibilizer Enhance the Properties of Carbon Fiber-Reinforced Composites?

**DOI:** 10.3390/polym15234608

**Published:** 2023-12-03

**Authors:** Prashant Gangwani, Mitjan Kalin, Nazanin Emami

**Affiliations:** 1 Laboratory for Tribology and Interface Nanotechnology, University of Ljubljana, 1000 Ljubljana, Slovenia; prashant.gangwani@fs.uni-lj.si; 2 Polymer-Tribology Group, Division of Machine Elements, Luleå University of Technology, 97187 Luleå, Sweden; nazanin.emami@ltu.se

**Keywords:** compatibilizer, carbon fiber, polymer composites, UHMWPE, PTFE, PPS

## Abstract

We have evaluated the effectiveness of compatibilizers in blends and composites produced using a solvent manufacturing process. The compatibilizers were two different types of polyethylene (linear low-density and high-density) grafted with maleic anhydride (MAH) and a highly functionalized, epoxy-based compatibilizer with the tradename Joncryl. The selected material combinations were an ultra-high-molecular-weight polyethylene (UHMWPE) with MAH-based materials as compatibilizers and a polyphenylene sulfide plus polytetrafluoroethylene (PPS-PTFE) polymer blend with an epoxy-based compatibilizer. The findings revealed that while the compatibilizers consistently enhanced the properties, such as the impact strength and hardness of PPS-based compositions, their utility is constrained to less complex compositions, such as fibrous-reinforced PPS or PPS-PTFE polymer blends. For fibrous-reinforced PPS-PTFE composites, the improvement in performance does not justify the presence of compatibilizers. In contrast, for UHMWPE compositions, compatibilizers demonstrated negligible or even detrimental effects, particularly in reinforced UHMWPE. Overall, the epoxy-based compatibilizer Joncryl stands out as the only effective option for enhancing mechanical performance. Thermal and chemical characterization indicated that the compatibilizers function as chain extenders and enhance the fiber–matrix interface in PPS-based compositions, while they remain inactive in UHMWPE-based compositions. Ultimately, the incompatibility of the compatibilizers with certain aspects of the manufacturing method and the inconsistent integration with the polymer are the main reasons for their ineffectiveness in UHMWPE compositions.

## 1. Introduction

Polymers are materials composed of repeating molecular structures, known for characteristics such as their light weight, self-lubricity, and versatility in shaping complex forms. These qualities have rendered them indispensable in various applications [1,2,3,4]. However, despite these benefits, they exhibit downsides such as low mechanical strength and poor thermal stability, especially when compared to traditional materials like metals. To alleviate these issues and have better control over the properties, fillers having dissimilar characteristics are incorporated to produce composites with superior properties [4,5].

Previous research in our group has indicated that a polymer like ultra-high-molecular-weight polyethylene (UHMWPE) and a blend of polyphenylene sulfide plus polytetrafluoroethylene (PPS-PTFE) with various fillers are excellent choices for water-lubricated tribological applications [6,7]. A challenge we observed is that the interactions between the added components and the polymer are not always ideal, especially if the polymer is non-polar. Popular strategies based on the available literature to address this include treating the components to increase their polarity [8,9] or introducing another component, such as a compatibilizer, whose primary function is to mediate the interactions between the original components, enhancing their bonding [10,11,12].

Compatibilizers, which have highly polar functional groups, can form physical or chemical bonds with polymer chains, leading to a more homogeneous blend when multiple base polymers are used [13,14]. They are also observed to be beneficial when combined with recycled thermoplastics [15,16]. A common mechanism of compatibilizers involves bonding one end to the polymer matrix and the other to the filler, promoting superior adhesion between the matrix and the filler [17,18]. The studies report enhancements in various properties when explored with blends [10,16,19] and fillers [11,12,20]. The improvements often relate to enhanced mechanical properties [10,11,12,16,20,21], with some studies also reporting improved viscoelastic properties [12,17]. The above advantages make compatibilizers a promising addition to polymeric composites. However, they can also have negative effects on properties such as crystallinity [11,12,19]. Although a decrease in crystallinity is commonly associated with a decline in the strength and stiffness of a neat polymer, this is generally not the case when considering the properties of the composite material. This is attributed to advances resulting from additional factors, such as the improved fiber–matrix interface or the miscibility in blends.

Several authors have investigated the incorporation of compatibilizers in different polymers reinforced with fibers [11,12,17,20]. Dumbaz et al. [12] conducted a study into the effect of various compatibilizers and their concentrations in polyphenylene sulfide (PPS) reinforced with carbon fibers. Their findings revealed an increased strength and storage modulus for all the tested compatibilizers, with the epoxy-based compatibilizer Joncryl-ADR-4300F (BASF; Ludwigshafen, Germany) exhibiting the best adhesion between the fibers and the polymer matrix. In a separate study [11], researchers utilized maleic anhydride (MAH)-based compatibilizers in a short-carbon-fiber-reinforced polypropylene system, observing enhancements in strength, hardness, and modulus but without compromising the thermal properties. In addition to improved interfaces between the fiber and the matrix, having polypropylene-grafted MAH compatibilizer also mitigated issues related to polymer miscibility. Investigations into polyethylene composites with MAH and glycidyl methacrylate compatibilizers were also conducted [17] and have demonstrated consistent improvements in the mechanical properties of all the compatibilizers tested. However, the manufacturing technique used in all the referenced studies involving compatibilizers [10,11,12,16,17,19,20,21] seems to be limited to extrusion or melt mixing, sometimes followed by injection molding. Studies with compatibilizers in other manufacturing processes, such as the solvent method, could not be found, and there was no information about the incorporation location or the effect of different manufacturing steps. Given the challenges posed by materials that are not extrusion compatible, such as those exhibiting a high blend viscosity, it is necessary to investigate these gaps.

This study looks at the effectiveness of compatibilizers in addressing recognized problems and potentially enhancing the properties of the previously mentioned materials utilized by our research group. This evaluation requires the fabrication of materials based on UHMWPE and PPS-PTFE using a solvent manufacturing method that has been optimized for parameters in previous research. The effect of the compatibilizer on the mechanical, surface, and thermal properties in different polymer/blend systems was explored. Furthermore, the influence of the manufacturing temperature and the compatibilizer’s incorporation on various properties were also investigated for selected systems.

## 2. Materials and Methods

### 2.1. Materials

Two different types of composites were prepared in this study, based on a UHMWPE matrix and PPS-PTFE blend matrix. The UHMWPE used in this study was GUR 4170 (Celanese; Irving, TX, USA) with a molecular weight of ~10.3 million and an average particle size of 120 µm (d50), and procured by Resinex (Logatec, Slovenia). The PPS and PTFE were both purchased from Sigma Aldrich (Product no. 182354 and 68096, respectively). 

**Figure 1:**
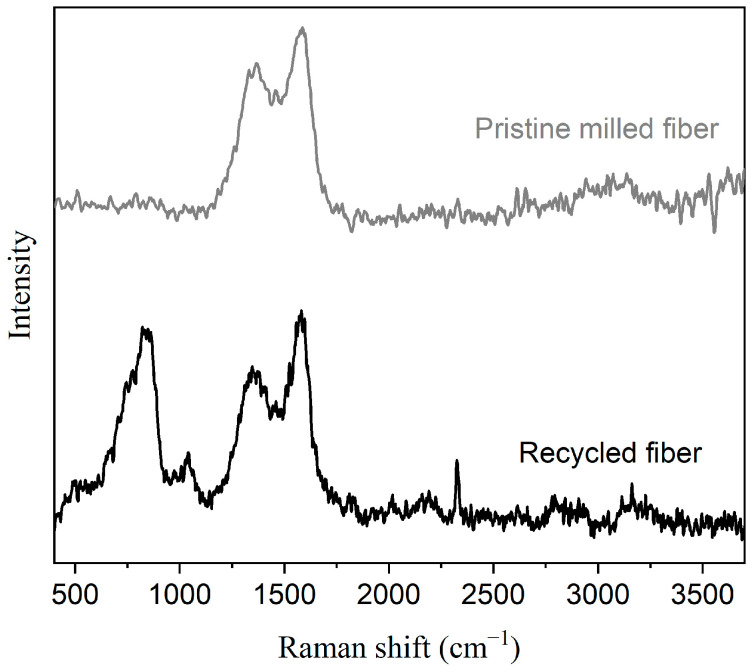
Raman spectra of recycled and pristine carbon fibers.

For the reinforcements, recycled short carbon fibers (CF.LS-MLD_100_) with an average length of 100 µm were used (Procotex S.A. corporation; Languidic, France). These fibers were found to have equivalent performance to pristine SCF in a non-polar matrix like UHMWPE [22]. The Raman spectra from these recycled fibers and pristine milled fibers of equivalent length are shown in Figure 1. The recycled fibers have additional, distinct peaks between 700 and 1250 and around 2300 cm^−1^. Unlike long carbon fibers, pristine short carbon fibers (SCFs) are not prepared using sizing agents such as PA or epoxy. This suggests that the recycled SCFs might have additional benefits over fresh fibers as a result of having potential groups on the surface, a consequence of the post-processing performed to recover them. PE-based compatibilizers, SCONA TPPE 1102 PALL (linear low-density polyethylene or LLDPE based) and SCONA TPPE 1212 PAHD (high-density polyethylene or HDPE based) were selected for use in UHMWPE-based composites. Both compatibilizers are highly maleic anhydride (MAH) functionalized and are manufactured by BYK (Chemie GmbH; Wesel, Germany). The MAH content in them is greater than 1.5%, and the recommended concentration with fibers is around 2–4%, according to information provided in the company datasheet. For the PPS-PTFE compositions, an epoxy-based compatibilizer, Joncryl 4468 from BASF (SE; Ludwigshafen, Germany), was selected and provided by the BTC Chemical Distribution Unit (Copenhagen, Denmark). 

### 2.2. Manufacturing 

**Figure 2:**
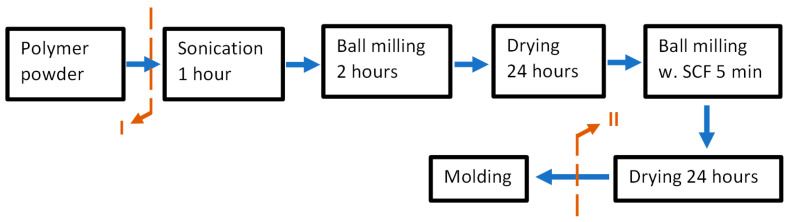
Flowchart showing composite manufacturing process with possible locations where compatibilizers can be included, marked as I and II.

The carbon fiber-reinforced composites were manufactured using the process shown in Figure 2. This process has been used for a long time by the group and is optimized for different parameters [6,23,24,25].

In the case of UHMWPE, there are two possible sites to incorporate the compatibilizers during the manufacturing process, denoted by I and II in Figure 2. In the case of the epoxy compatibilizer for the PPS-PTFE composites, only location II is viable due to the low glass-transition temperature of the compatibilizer. When trying location I for the epoxy compatibilizer, the sonication step caused the temperature to rise above 60 °C, which in turn caused the compatibilizer to soften and settle down. For the UHMWPE composites, both positions were tried and denoted in the subscript of the nomenclature, where the compatibilizer is added in the process.

Manufacturing processing: The polymer powder or polymer blend was sonicated in ethanol for 1 h, followed by ball milling at 200 rpm for two hours. The milling was in a PM 100 planetary ball mill (Retsch GmbH; Haan, Germany) using 5 mm zirconia balls. The powder or blend mix in slurry form was then dried at 70 °C for 24 h. Following this, SCF was added to the dried powder and ball milled for homogenization. The milling in this step was kept to only 5 min and at a low speed of 100 rpm. The parameters used in both milling operations were optimized in a previous study to limit the distortion of the polymer and breaking of the fibers [26]. Finally, the dried composite powder was consolidated into bars using compression molding for the UHMWPE composites and mini-injection molding for the PPS/PTFE composites. For the compression molding, the plates were pressed at 15 MPa and 190 °C for 1 h, followed by cooling at 5 °C/min. This involved an LZT-UK-35-L Laboratory press (Langzauner GmbH; Lambrechten, Austria). The consolidation method using compression molding was kept same as in previous studies by the group used for manufacturing nano-composites [7,25,26]. Although the previous studies suggested no degradation from the selected molding parameters, an FTIR analysis was conducted on a molded part to ensure the absence of peaks at ~1715 cm^−1^, which typically denotes oxidation due to elevated temperatures [27]. A HAAKE™ MiniJet Pro Piston (Thermo Fisher; Karlsruhe, Germany) injection-molding machine was used to consolidate the PPS/PTFE blend composites. The parameters used for injection molding are listed in Table 1. These selections are similar to those used in other studies by the group [6,23,24], except for the cylinder temperature. The samples were kept inside the mold until the mold temperature reached 90 °C to ensure the crystallization process was completed.

### 2.3. Compositions 

To study the efficacy of the compatibilizers for the selected materials in the presented manufacturing process, the following composites, listed in Table 2, were manufactured. The concentration of SCF in all the composites was 10 wt.% when included, and the ratio of the PPS/PTFE blend was 60–40 wt.%. This specific ratio of PPS/PTFE blend was selected based on previous optimization studies performed by the group [7,23]. All the concentrations are in weight percentages.

### 2.4. Experimental 

Mechanical performance: The composites were first characterized for properties such as microhardness and impact strength to determine the effect of the compatibilizer on the mechanical properties. To determine the hardness, Vickers measurements were conducted using a diamond cone as the indenter on a Duramin AC40 instrument (Struers; Ballerup, Denmark). The hardness (HV) values were determined using the following equation:

(1) $$\mathrm{HV}=k\;\times \;\frac{P}{d^{2}}\;\times \;10^{6}$$

where d is the average diagonal length of the indentation (mm) formed after applying a load P (N) and k is a known geometrical factor.

An average of ten measurements made with a 10 s dwell time was considered for the hardness. The force for determining the hardness depended on the composite under test, i.e., 100 gmf was sufficient to produce indentations in the UHMWPE composites, and 500 gmf was required in the case of the PPS/PTFE compositions. 

Impact strength measurements were conducted according to the ISO standard for impact properties of plastics [28]. The samples had dimensions of 80 × 10 × 4 mm^3^. Three tests were performed for the average of the impact strength. 

Wettability: The contact angle was measured to investigate the hydrophobicity of the samples. These measurements were made with an Attension Theta tensiometer (Biolin Scientific; Västra Frölunda, Sweden), an optical goniometer. We used distilled water, with a drop volume of around 4 µL deposited on the sample surface. 

Thermal characterization: Differential scanning calorimetry (DSC, Mettler Toledo; Columbus, OH, USA) was used to measure the degree of crystallinity and the melting temperature for the materials. Sample weights of around 10 mg were used in a standard alumina crucible of 40 µL. A nitrogen gas flow of 80 mL/min was maintained for all the measurements. The heating and cooling rates are both kept at 10 °C/min. The crystallinity of the compositions was determined from the melting peaks and using the following equation:

(2) $$X_{c}=\frac{\;\;\;\Delta H\;\;\;\;}{{\;\;Mc\times \Delta H}_{100}}\times 100\%\;\;\;\;\;\;\;\;$$

where

ΔH is the enthalpy of fusion determined by measuring the area under the melting peak.
ΔH_100_ is the enthalpy of fusion of 100% crystalline polymer.
Mc is the fraction of polymer (in wt. %) whose crystallinity is measured in the composition.

Scanning electron microscopy: The samples tested for impact strength were observed in a JSM IT100 scanning electron microscope, SEM (JEOL; Akishima, Japan) to examine the polymer–reinforcement interface. This was conducted under high-vacuum conditions, with the samples coated with gold prior to the observation. Energy-dispersive X-ray spectroscopy (EDS, JEOL; Akishima, Japan) studies were conducted in addition to the SEM. Element mapping was used to highlight the presence of different chemical elements and their distribution.

Fourier-transform infrared spectroscopy (FTIR): FTIR was conducted for selected compositions (pre- and post-consolidation). This was conducted in attenuated-total-reflection (ATR) mode on a Nicolet Summit spectrometer (Thermo Fisher Scientific; Karlsruhe, Germany). The spectra were generated from 16 consecutive scans at a resolution of 1 cm^−1^ with the sample pressed against the crystal under constant load.

## 3. Results

### 3.1. Mechanical Testing

**Figure 3:**
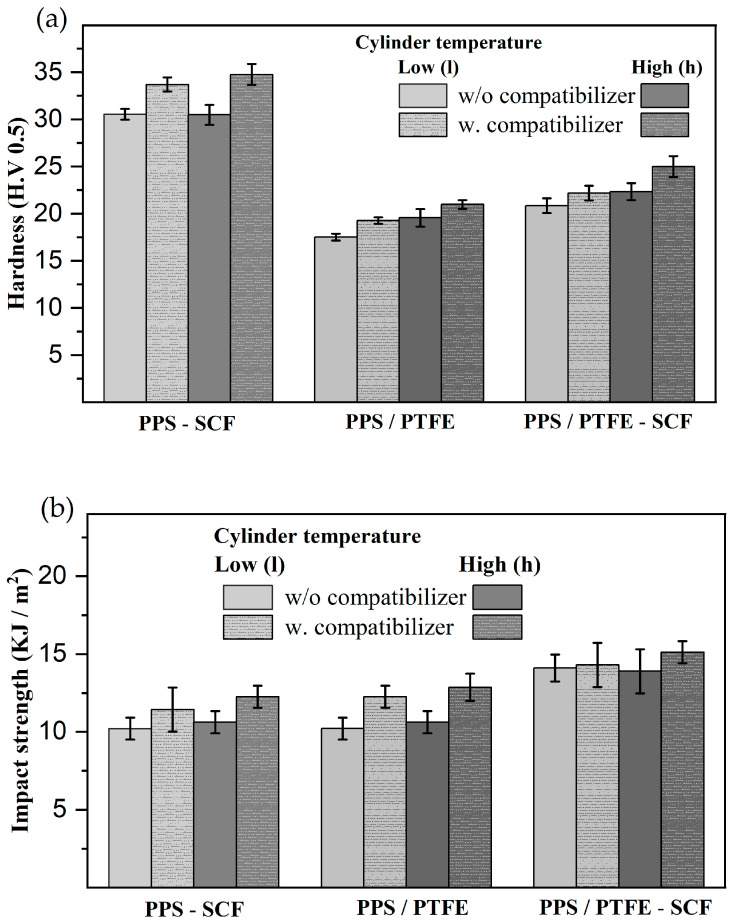
Effect of 1.5 wt.% compatibilizer and manufacturing temperature on (**a**) hardness and (**b**) impact strength for PPS/PTFE compositions.

The hardness and impact results for the PPS/PTFE-based compositions are presented in Figure 3. The PPS-SCF compositions have the highest hardness values (30–35 HV), followed by the PPS/PTFE-SCF compositions (20–25 HV), with the lowest being the PPS/PTFE blend (17–20 HV). Conversely, when considering the impact strength, the PPS/PTFE-SCF compositions show the highest values (14–15 KJ/m^2^), while the PPS-SCF and PPS/PTFE blends have overlapping values (10–13 KJ/m^2^). Enhancements to the mechanical properties were observed for all the PPS/PTFE compositions with a 1.5 wt.% epoxy compatibilizer. Adding a compatibilizer to the PPS-SCF composites and PPS/PTFE blend improved the hardness by 10–13%. At the same time, the impact strength increase due to the compatibilizer was more significant for the PPS/PTFE blend, around 20%, compared to as much as 15% for the PPS-SCF composition. As for PPS/PTFE-SCF compositions, a similar improvement in hardness was observed, but no significant effect on the impact strength was noted due to the compatibilizer.

**Figure 4:**
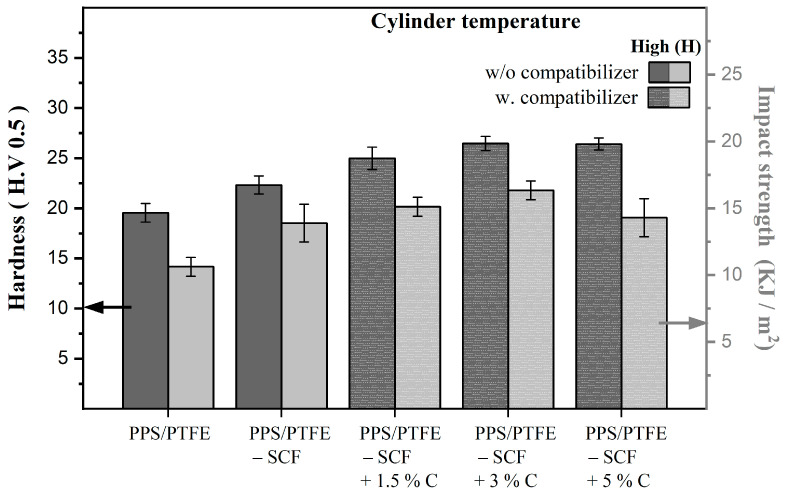
Effect of compatibilizer concentration on hardness and impact strength of PPS/PTFE-SCF composites manufactured at 375 °C.

Another crucial consideration for the PPS/PTFE compositions is the effect of manufacturing temperature. In the case of the PPS-SCF compositions, the manufacturing temperature had a negligible impact, as the compositions prepared at both cylinder temperatures (315 °C and 375 °C) had similar hardness, regardless of the presence of a compatibilizer. However, for the PPS/PTFE blends and the PPS/PTFE-SCF compositions, a 10% increase in hardness was observed when the materials were produced at a higher temperature of 375 °C. Concerning the impact strength, there was a marginal but consistent 5–10% improvement for the PPS-SCF compositions and the PPS/PTFE blends when manufactured at a higher temperature. For the PPS/PTFE-SCF composites, there is no significant difference due to the manufacturing temperature on the impact strength values. Figure 4 illustrates the effect of the compatibilizer concentration on the hardness and impact strength of the PPS/PTFE-SCF composition. It is evident that both the hardness and the impact strength initially improve with an increase in the compatibilizer concentration from 0 to 3 wt.%. However, when the compatibilizer concentration reaches 5 wt.%, it has no further effect on the hardness and even a negative effect on the impact strength, reducing the values.

**Figure 5:**
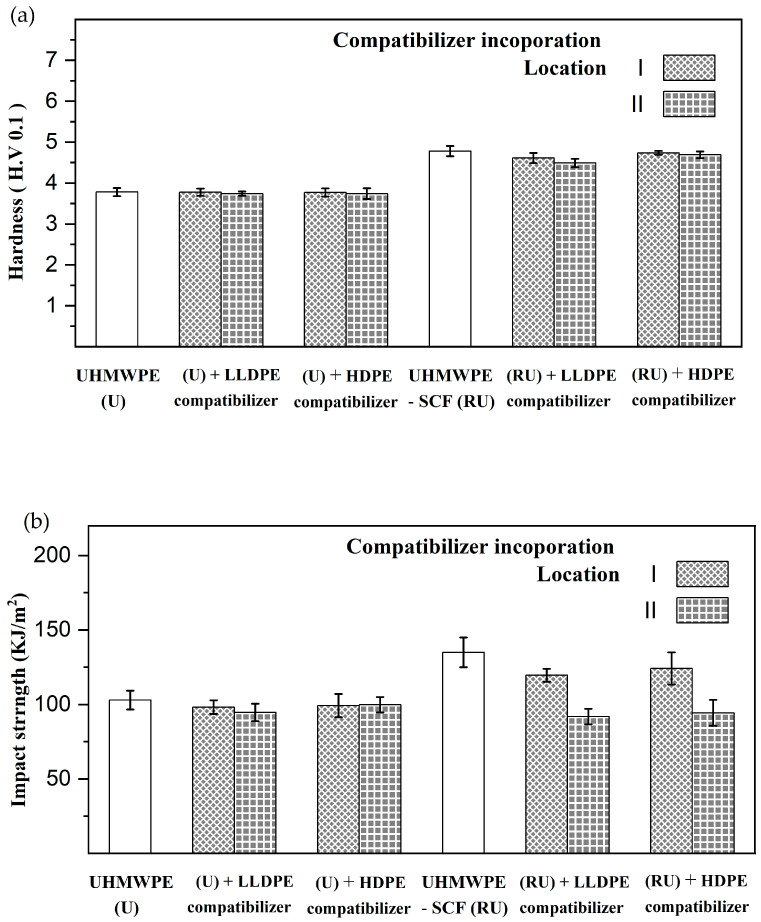
Effect of 3 wt. % HDPE- and LLDPE-based MAH compatibilizers on (**a**) hardness and (**b**) impact strength of UHMWPE-based compositions.

For the case of the UHMWPE compositions, the hardness and impact strength results are presented in Figure 5. The UHMWPE-SCF compositions had a higher hardness (4.5–4.8 HV), followed by the unreinforced compositions (~3.7 HV). The impact strength has a similar trend, where the UHMWPE-SCF compositions had higher values (100–135 KJ/m^2^) in comparison, but the lower range coincides with that of the unreinforced UHMWPE compositions (~100 KJ/m^2^). With the introduction of the compatibilizer, enhancements in either property were not observed, irrespective of whether the compatibilizer was HDPE- or LLDPE-based. Rather, both compatibilizers had either no effect or had a detrimental effect on these properties, especially on the impact strength. In compositions lacking SCF, the reduction in impact strength was less significant (below 8%), with the compatibilizer type and the location of the incorporation having a marginal effect. For the UHMWPE-SCF compositions, on the other hand, the reduction in impact strength was more pronounced (up to 30%) after the introduction of the compatibilizer. When comparing the two compatibilizer options, the difference was relatively small, with the HDPE having slightly better performance than the LLDPE-based compatibilizer. Moreover, the incorporation location was observed to influence the impact strength, with a further reduction (20% less) in performance observed when the compatibilizers were incorporated at location II. A less critical but similar trend was also observed for the hardness, where the deterioration was again less significant in the absence of reinforcements. Furthermore, for the UHMWPE-SCF compositions, the incorporation location II again exhibited a marginally lower hardness (less than 5%) in comparison to location I.

### 3.2. Wettability

**Figure 6:**
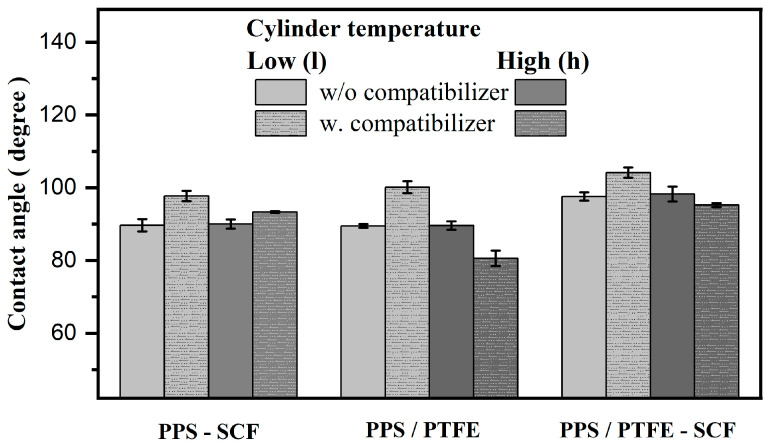
Effect of 1.5 wt.% compatibilizers and manufacturing temperature on contact angles.

The contact angles for the PPS-based compositions are shown in Figure 6. In the absence of a compatibilizer, the contact angles for the PPS-SCF composites and PPS/PTFE blend were around 90°, while for the PPS/PTFE-SCF, they were higher (around 98°). Furthermore, these values remain unchanged, irrespective of the composite’s manufacturing temperature. 

With the inclusion of the compatibilizer, an increase in the value was observed for the compositions manufactured at a lower temperature. The highest value was demonstrated by the PPS/PTFE-SCF composition with a compatibilizer. However, an increase was observed for all the compositions, with the following trend:

$$\frac{\mathrm{PPS}/\mathrm{PTFE}-\mathrm{SCF}\;\mathrm{composite}\;(104{}^{\circ})\;>\;\mathrm{PPS}/\mathrm{PTFE}\;\mathrm{Blend}\;(100{}^{\circ})\;>\;\mathrm{PPS}-\mathrm{SCF}\;\mathrm{composite}\;(97.5{}^{\circ})}{\begin{array}{c} ↓\\ With\;compatibilizer\;(315\;{}^{\circ}C) \end{array}}$$

The opposite was observed for the composites manufactured at higher temperatures with compatibilizers, i.e., all the compositions showed a decrease in the contact angle. The decrease was less apparent with the SCF and more severe when the PTFE was present. The PPS/PTFE-SCF composition appeared to have a smaller drop in value in comparison to the PPS/PTFE blend. The following trend was observed for the 375 °C manufacturing temperature:

$$\frac{\mathrm{PPS}-\mathrm{SCF}\;\mathrm{composite}\;(95.2{}^{\circ})\;>\;\mathrm{PPS}/\mathrm{PTFE}-\mathrm{SCF}\;\mathrm{composite}\;(93.3{}^{\circ})\;>\;\mathrm{PPS}/\mathrm{PTFE}\;\mathrm{Blend}\;(80.5{}^{\circ})\;}{\begin{array}{c} ↓\\ With\;compatibilizer\;(375\;{}^{\circ}C) \end{array}}$$

Further, the effect of the compatibilizer concentration on the contact angles for the PPS/PTFE-SCF composites manufactured at 375 °C was as follows:

1.5% C (95°) < 0% C (98°) ≈ 3% C (98°) < 5% C (100°).

A decrease in the contact angle was noted with the initial inclusion, and a further rise was observed with an increasing concentration of the compatibilizer. However, both changes are only marginal in comparison. 

For the UHMWPE compositions, in the case of neat UHMWPE, a contact angle of 85° was observed, and for the UHMWPE-SCF composite, around 94° was observed. No noticeable difference was observed either with the incorporation of any compatibilizer or the inclusion location. 

### 3.3. Thermal Characterization

Table 3 shows a summary of the thermal properties measured by DSC for selective PPS/PTFE-SCF compositions. Manufacturing and processing parameters are important factors that could affect the degree of crystallinity in polymer-based materials. To differentiate the impact from processing and thermal history in the manufactured samples, both the first and second heat cycles are presented for the crystallinity. In the case of the PPS, it is apparent that the melting temperature (Tm) remains consistent, regardless of the presence of compatibilizers or the different manufacturing temperatures in the injection-molding cylinder. However, the crystallinity is noticeably reduced when compatibilizers are introduced, with a 10% decrease observed during the first heating cycle and a 6% reduction during the second heating cycle under both temperature conditions. With the addition of PTFE to the PPS matrix, the presence of the compatibilizer appears to result in a marginally higher crystallinity for the PTFE. Furthermore, during the first heating cycle, materials manufactured at 315 °C exhibit a roughly 6% higher crystallinity than those processed at 375 °C. However, it is worth noting that the values from the second heating cycle indicate the absence of any effect of the manufacturing temperature on the PTFE’s crystallinity.

**Figure 7:**
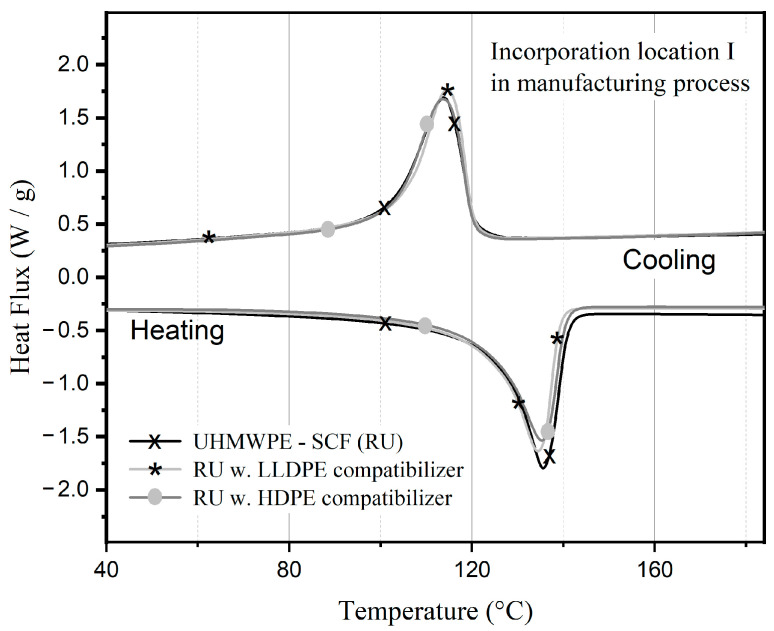
DSC curves of UHMWPE/SCF compositions manufactured with compatibilizers incorporated at location I.

The DSC data for UHMWPE-SCF compositions manufactured with both compatibilizers at location I are shown in Figure 7. There are no noticeable shifts observed in either the heating or cooling cycle, with the Tm around 135 °C. Additionally, the calculated crystallinity values are not significantly different, at approximately 48%, across all compositions.

### 3.4. Scanning Electron Microscopy

**Figure 8:**
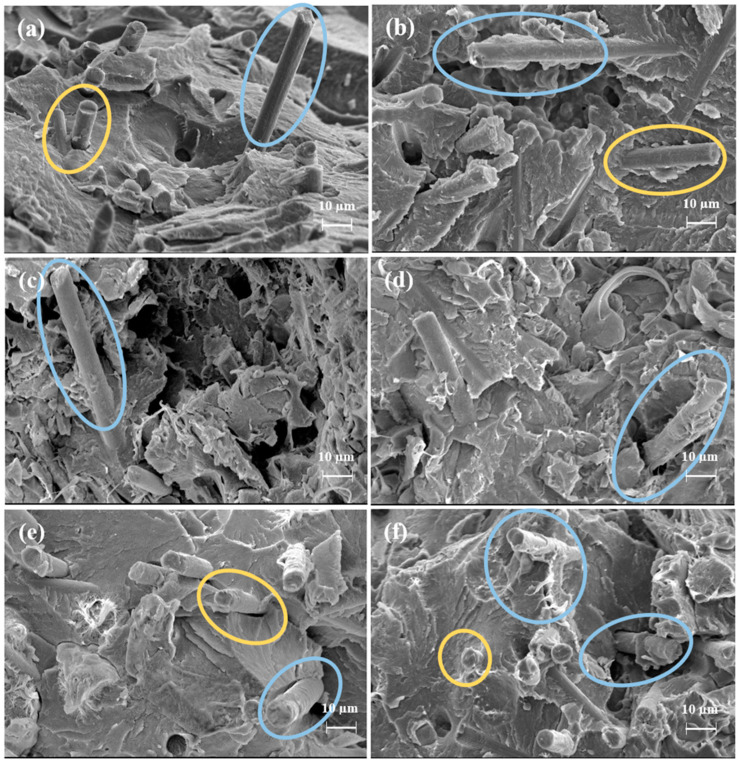
SEM images of PPS-SCF composition at 315 °C (**a**,**b**); PPS/PTFE-SCF compositions manufactured at 315 °C (**c**,**d**) and 315 °C (**e**,**f**) without and with compatibilizers (**left** vs. **right**).

The SEM images of the fracture surface of the impact samples are shown in Figure 8 for the PPS-SCF and PPS/PTFE-SCF composites, in the absence (Figure 8a,c,e) and presence (Figure 8b,d,f) of compatibilizers. These fractured surfaces exhibit distinct signs that are indicative of brittle fractures, characterized by sharp edges and deep valleys in the case of the PPS-SCF compositions (Figure 8a). Moreover, a lack of adequate adhesion is apparent at the interface between the reinforcement and matrix surfaces, highlighted by the yellow zones. Additionally, in the blue zones on the outbound fibers’ surface, there is minimal-to-no observable polymer adherence. In the PPS/PTFE-SCF compositions (Figure 8c,e), the previously mentioned features associated with brittle fractures and poor fiber–matrix adhesion persist but are abated to some extent. Nonetheless, the surface of the outbound fibers remains devoid of any attached polymer material. In compositions incorporating compatibilizers, a more homogenous and level surface is observed compared to their counterparts lacking compatibilizers. Moreover, the fibers appear to be better gripped, and a greater adherence of the polymer on the exposed fiber surface is noted.

**Figure 9:**
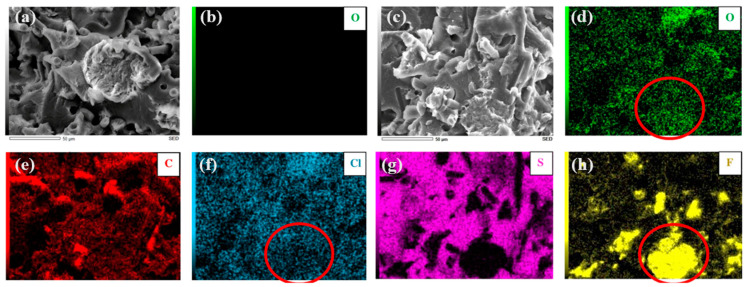
SEM images with respective EDS mapping for PPS/PTFE-SCF composition manufactured at 375 °C without compatibilizer (**a**,**b**) and with compatibilizer (**c**–**h**).

Figure 9 presents the EDS element mapping for the PPS/PTFE-SCF composition. When examining composites manufactured without a compatibilizer (Figure 9a), oxygen is lacking. Additionally, Figure 9d–h provides EDS mapping for all the other observed elements. These elements, i.e., carbon, oxygen, and chlorine, exhibit a uniform distribution, with several similarities noted between the latter two. In contrast, sulfur appears to have locations where it is noticeably absent, while a high intensity of fluorine is observed at many of these locations, as indicated by the red region. In this region, the intensity of the oxygen intensifies, even though the chlorine appears to decline.

### 3.5. Fourier-Transform Infrared Spectroscopy

**Figure 10:**
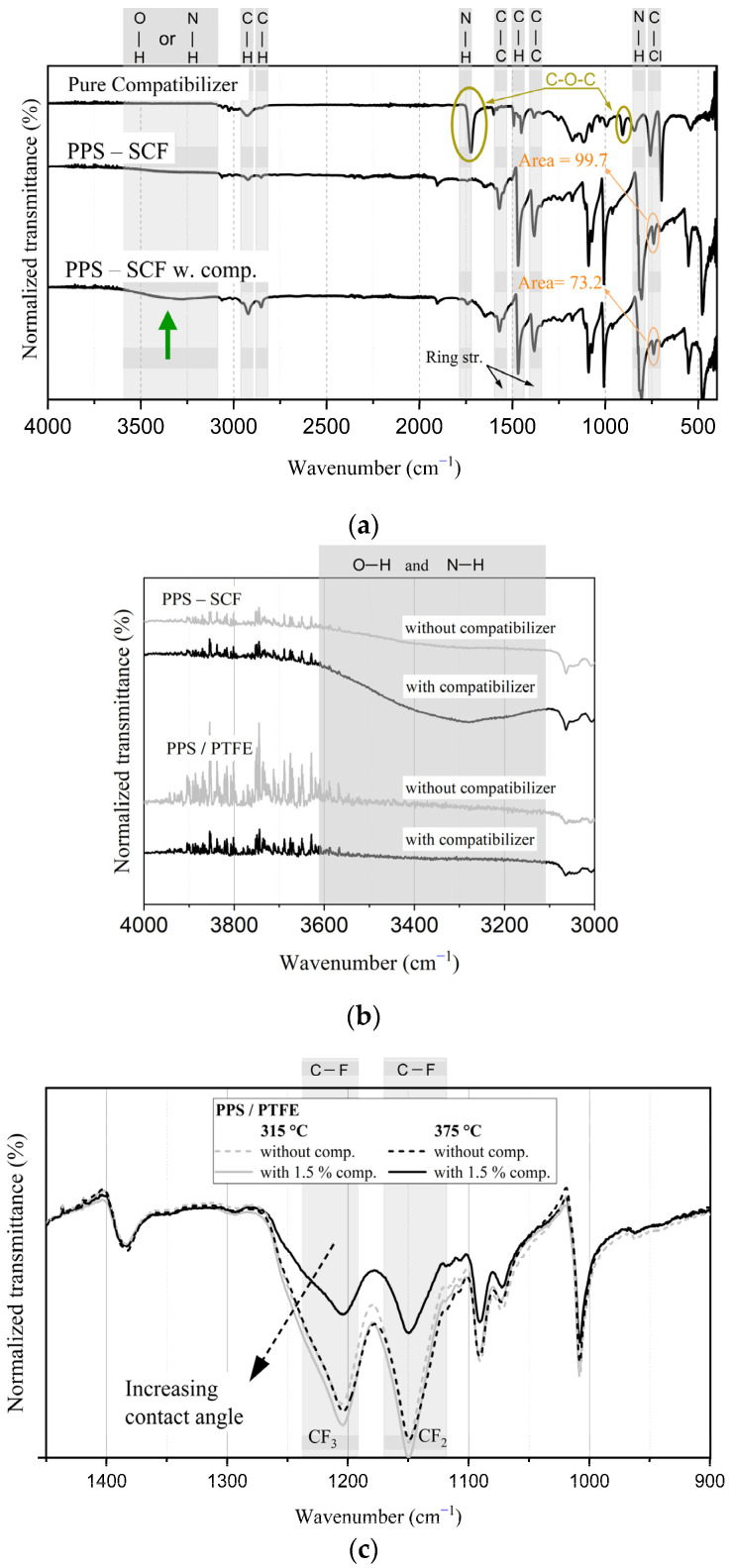
FTIR spectra of (**a**) compatibilizer and PPS composites manufactured at 375 °C cylinder temperature over complete wavenumber range. (**b**) PPS-SCF and PPS/PTFE compositions focusing on 4000–3000 cm^−1^ range. (**c**) PPS/PTFE composites manufactured at different cylinder temperatures focusing on 1450–900 cm^−1^ range.

The FTIR spectra for the PPS/PTFE-based compositions are shown in Figure 10. Features such as the benzene-ring vibration of the PPS (at 1382, 1548, and 1572 cm^−1^), the -C-Cl stretching peaks (at 704 and 741 cm^−1^), and the alkanes -CH_2_- (1470, 2850, 2925 cm^−1^) appear for all the compositions, irrespective of the manufacturing temperature. In the SCF-reinforced compositions, additional peaks at 806, 1572, 3300 cm^−1^, and 1000–1300 cm^−1^ are also observed. Moreover, the key peaks from the compatibilizer at 907 and 1725 cm^−1^ (denoting epoxy and carbonyl stretching groups) are not observed in the composites containing compatibilizer. Upon normalization, the area of the peak under 741 cm^−1^ is reduced (25% with respect to its non-compatibilizer counterpart) and shifted slightly to the right when the compatibilizer is present. Additionally, the broad absorbance band observed between 3100 and 3600 cm^−1^ has a higher intensity for the PPS composite containing compatibilizer. Furthermore, a distinct peak at 1747 cm^−1^ is observed in the composite containing compatibilizer, suggesting N-H bending.

Upon further assessment of the spectra, a distinction was noted on the high-wavenumber side. As shown in Figure 10b, a wide absorbance peak for reinforced PPS compositions around 3300 cm^−1^ is absent in the PPS/PTFE blend, irrespective of the compatibilizer’s presence. Moreover, the intensity of this peak appears to increase in the presence of compatibilizer. 

Moreover, differences are noted in the CF_2_ and CF_3_ absorption-peak intensities, which depend on both the compatibilizer and the manufacturing temperature. As observed for PPS/PTFE blends in Figure 10c, in the absence of a compatibilizer, the absorption peaks have similar intensity, and the manufacturing temperature has no effect. However, upon incorporating compatibilizers, the intensity of the peaks is marginally higher for the sample manufactured at 315 °C and reduced for the one manufactured at 375 °C.

**Figure 11:**
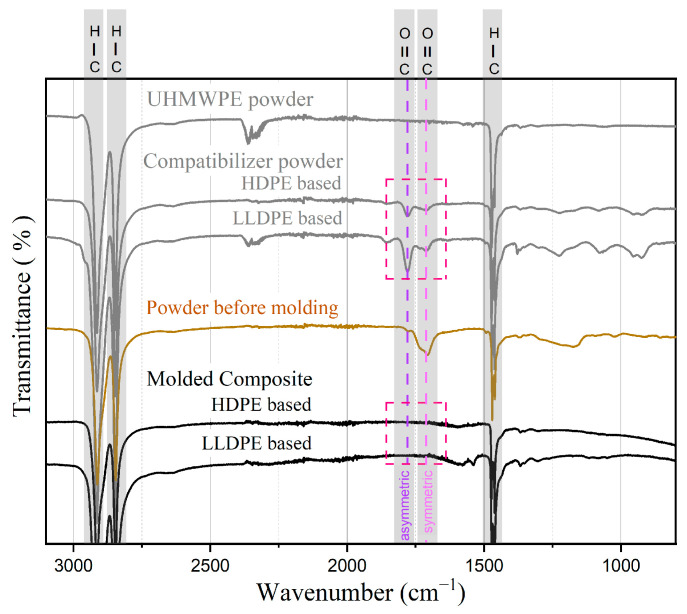
FTIR spectra for UHMWPE and compatibilizer powders and resulting composites.

For the case of the UHMWPE composites, the spectra are presented in Figure 11. High-intensity peaks for alkanes at 1470, 2850, and 2925 cm^−1^ are observed. Both compatibilizer powders also have extra peaks between 1718 and 1790 cm^−1^ (see Figure 11) that do not appear in the pure UHMWPE powder or in the final composites after compression molding. These peaks mark the stretching of the carbonyl group (-CO-) present in the compatibilizer powder. The intensity of the peak located at 1790 cm^−1^ is higher than the peak at 1718 cm^−1^ in both compatibilizers. Another spectrum of powder with the compatibilizer incorporated at location I was taken just before the molding step. In this case, the intensities of the above-mentioned peaks were reversed. Moreover, two additional peaks are observed in the final composites, at 1539 and 1577 cm^−1^, irrespective of the compatibilizer used.

## 4. Discussion

The hardness, impact strength, and contact angles of pure PPS are reported to be approximately 27.5 HV, 2.5 KJ/m^2^, and 83°, respectively [6,29]. The values for reinforced PPS (PPS-SCF) showed an increase for all of the above properties. The hardness, impact strength, and contact angles of the reinforced PPS compositions were 10%, 300%, and 7° higher in comparison to pure PPS. This increase in hardness and strength can be attributed to the reduced plastic deformation and energy absorption upon impact by the incorporated fibers [30,31]. Additionally, the hydrophobic nature of these fibers plays a role in increasing the contact angle of the composite material [6,32]. In the case of the blend (PPS/PTFE), a 300% increase in impact strength in comparison to pure PPS and around a 6° higher contact angle was again noted, aligning with the enhanced toughness and hydrophobic nature of the PTFE [29]. The hardness of the blend, however, was lower than the pure PPS (~30% lower), attributed to the minuscule hardness of the PTFE in comparison to the PPS. Furthermore, similar to the PPS, the inclusion of reinforcement in the blend (PPS/PTFE-SCF) also resulted in an increase in all values. The final increase in impact strength, in the absence of a compatibilizer, was 460% higher in comparison to pure PPS, while the increase in the contact angle was 17°. An increase in the hardness compared to the unreinforced blend was also noted; however, the hardness was still ~22% lower in comparison to pure PPS. 

In the absence of compatibilizers, the manufacturing temperature influenced the mechanical performance only in the presence of PTFE in the composition. This effect stemmed from the melting temperature of PTFE falling between the two different manufacturing temperatures in the injection-molding cylinder. SEM images (Figure 8) revealed that at lower temperatures, fragments of polymer were observed, which was not the case for the materials manufactured at higher temperatures. This suggests a more homogeneous blend of the two polymers at higher temperatures, resulting in an improved strength of the produced composite.

With the introduction of compatibilizers, an improvement in hardness and impact strength for the PPS-PTFE compositions was noted. This improvement with compatibilizers makes PPS-based composites more useful in already-established applications where resistance to impact loading and durability are key requirements, such as gears or bearings in industrial machinery and insulating components in electronic systems. Moreover, the ability to modify the wettability of these materials by adjusting the manufacturing temperature in the presence of compatibilizers further expands their utility in applications like pump and valve components.

In compositions containing SCF, the SEM analysis indicated improvements in the characteristics such as the fiber–matrix interface and wetting (chemical bonding) with the inclusion of the compatibilizers. Additionally, the EDS analysis (Figure 9) revealed the presence of oxygen in the composites containing compatibilizers, as expected due to the epoxy-based nature of the compatibilizer and the anticipated presence of oxygen in the reaction structures [33]. Furthermore, the presence of oxygen at locations with low chlorine intensity suggested that the compatibilizer was evenly distributed, not just around the PPS-based composites region.

Moreover, the disappearance of key peaks associated with compatibilizers, such as epoxy (907 cm^−1^) and carbonyl stretching (1725 cm^−1^), indicated that the compatibilizers were largely consumed during the manufacturing. Furthermore, the reduced crystallinity and a decrease in the -CCl stretching peaks at 741 cm^−1^ observed for the PPS in compositions containing compatibilizers support their function as chain extenders [12]. The resulting structures with compatibilizers were expected to have higher molecular weight, longer chains, and nested structures in the middle, as the compatibilizers were heavily branched [12,34]. Molecules with higher molecular weight and branched structures typically have lower crystallinity [35,36,37] without affecting the melting peaks (unless dealing with low molecular weights [38]). This explains the observed enhancement in hardness and impact strength with compatibilizers in blends, as the above properties are known to be related to molecular weight and crystallinity [39,40,41]. 

For the SCF-reinforced compositions (PPS-SCF and PPS/PTFE-SCF), the FTIR analysis revealed the presence of amine (-R-NH_2_) absorption peaks at 806, 1572, and 3300 cm^−1^, as depicted in Figure 10a. Furthermore, upon the addition of compatibilizers, a significant decrease and broadening of the absorption band for 3750–3250 cm^−1^ in SCF-containing compositions were observed. Consequently, it can be inferred that N-H stretching arising from secondary amine (-R2NH) [42] and the formation of -OH products [33] resulted from the reaction between epoxy groups with -R-NH_2_ on fiber and -SH chain end from PPS contribute to this wide absorption peak. This observation aligns with the improved fiber–matrix interface observed in the SEM images when compatibilizers were present.

While the use of higher manufacturing temperatures led to improved properties, it also resulted in a reduction in the crystallinity for PTFE when compatibilizers were introduced. Additionally, as indicated in Figure 10c, based on the intensity of the CF_2_ and CF_3_ absorption peaks, it is evident that the decomposition of PTFE occurred. PTFE is known to gradually decompose, releasing fluorine at temperatures exceeding 260 °C [43,44]. This explains the observed decrease in hydrophobic properties and suggests an accelerated degradation of PTFE at higher manufacturing temperatures. 

All of the factors mentioned above, coupled with the enhanced homogenization of the blend for materials manufactured at 375 °C, help elucidate the observed trend in the results. However, it is crucial to note that the substantial improvement in properties was only evident in the case of the reinforced PPS and PPS/PTFE blends, whereas it was merely marginal in the reinforced PPS/PTFE compositions. The primary reason being the reduced concentration of available PPS for the reaction, thus limiting its ability to manifest significant improvements in performance. The concentration variation indicated that a lack of a compatibilizer could not be attributed to this, as a decline in properties was observed when the concentration was increased, possibly from extensive curing of the epoxy, as has been noted in previous studies [33]. 

When analyzing the FTIR spectra for UHMWPE (Figure 11), it became apparent that the carboxyl groups within the MAH-based compatibilizers were consumed during the manufacturing process and consequently did not appear in the final composite after molding. However, no discernible peaks are observed around 1260 cm^−1^, signifying the absence of beneficial -C-O-C- linkages [45]. This absence is not accompanied by any improvements in performance or adhesion attributed to the presence of compatibilizers. The peak at 1790 cm^−1^ corresponds to the asymmetric stretching modes of carbonyl (C=O) in saturated MAH, which is responsible for its polar behavior [42]. Another band at 1718 cm^−1^ corresponds to the symmetric stretching of carbonyl (C=O). As Figure 11 illustrates, even before the final molding step, the peak associated with polarity appears to diminish while the intensity of the second band, indicative of symmetric stretching, increases. This suggests that the compatibilizer loses its polarity, likely explaining the absence of any improvement. Further investigations were conducted to bring attention to which component of the manufacturing process affected the polarity. Spectra of powders were generated after each step, leading to the conclusion that the solvent used, ethanol, was responsible for reducing the polarity.

**Figure 12:**
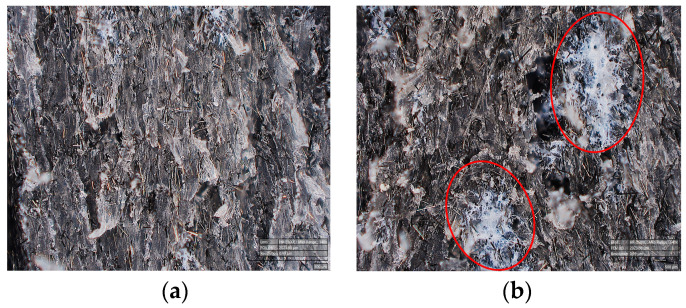
Micrographs of UHMWPE composites with LLDPE compatibilizers incorporated at (**a**) location I and (**b**) location II in the manufacturing process.

On the other hand, materials produced with incorporation at location II did not exhibit unintended interactions with the solvent. The micrographs of the impact samples (Figure 12) offer additional insights into the observed dependence on the incorporation location. As evident from the micrograph (Figure 12b), the highlighted red zones signify material agglomeration at specific sites for the sample with incorporation location II. The absence of such regions in the impact samples with a composition lacking a compatibilizer suggests a correlation with the presence of a compatibilizer in the material. Furthermore, no such sites with agglomerated material were observed for the incorporation location I (Figure 12a), indicating a dependency on the incorporation location. All the aforementioned findings suggest that the compatibilizers agglomerated when incorporated at location II, thereby not contributing to any enhancements. Hence, it can be reasonably inferred that compatibilizers did not effectively function with our selected manufacturing method.

## 5. Conclusions

To assess the potential to enhance the properties of PPS-PTFE- and UHMWPE-based composites, compatibilizers were introduced into the well-established solvent manufacturing process. The following key observations were made:

- In the case of PPS-based compositions, compatibilizers proved to be effective primarily in less-complex compositions, like the PPS-PTFE polymer blend or short-carbon-fiber-reinforced PPS, with compatibilizers delivering 10% higher hardness and 20% greater impact strength. For more intricate compositions, such as the short-carbon-fiber-reinforced PPS-PTFE, the improvements were only marginal and failed to justify the use of compatibilizers. Interestingly, neither the high manufacturing temperature nor the concentration of the compatibilizer posed severe problems. Instead, the limitation appeared to be the availability of the polymer to react, which hindered the effectiveness of the compatibilizers.
- In the UHMWPE compositions, the compatibilizers either had no effect or, in some cases, had a detrimental impact on the properties. This was attributed to their incompatibility with certain elements of the manufacturing method and their inability to consistently integrate into the material.

## Figures and Tables

**Table 1:** Parameters for injection molding.

Parameter	Value
**Pressure**	
Pressing	500 bar
Post pressing	300 bar
**Temperature**	
Cylinder	315 or 375 °C
Mold	200 °C
**Time**	
Injection	45 s
Hold	15 s

**Table 2:** UHMWPE- and PPS/PTFE-based composites.

Matrix	Additives
PPS-SCF	-
	+1.5% Epoxy comp.
PPS/PTFE	-
	+1.5% Epoxy comp.
PPS/PTFE-SCF	-
	+1.5% Epoxy comp.
	+3% Epoxy comp.
	+5% Epoxy comp.
UHMWPE	-
	+3% HDPE or LLDPE comp.
UHMWPE-SCF	-
	+3% HDPE or LLDPE comp.

**Table 3:** Properties from DSC for PPS/PTFE compositions.

Property	PPS/PTFE-SCF at 315 °C		PPS/PTFE-SCF at 375 °C	
	No Comp.	1.5 wt.% Comp.	No Comp.	1.5 wt.% Comp.
PPS				
1st heating				
Tm (°C)	283	282	285	282
Crystallinity (%)	61.8	51.8	59.5	53.2
2nd heating				
Tm (°C)	280	277	279	277
Crystallinity (%)	54.8	48.1	53.4	47.5
PTFE				
1st heating				
Tm (°C)	327	327	326	326
Crystallinity (%)	75.2	79.1	70.3	72.9
2nd heating				
Tm (°C)	328	327	328	327
Crystallinity (%)	71.1	75.2	72.0	76.9

## Data Availability

Data are contained within the article.
